# Evaluation of a follow-up programme after curative resection for colorectal cancer

**DOI:** 10.1038/sj.bjc.6990049

**Published:** 1999-01

**Authors:** J D Howell, H Wotherspoon, E Leen, T C Cooke, C S McArdle

**Affiliations:** University Department of Surgery, Glasgow Royal Infirmary, Glasgow, G31 2ER, UK; University Department of Surgery, Western General Hospital, Edinburgh, EH4 2XU, UK

**Keywords:** colorectal cancer, follow-up, liver metastases

## Abstract

Frequent liver imaging can detect liver metastases from colorectal cancer at an asymptomatic stage. © 1999 Cancer Research Campaign


*British Journal of Cancer* (1999) **79**(2), 308–310
© 1999 Cancer Research Campaign

### Evaluation of a follow-up programme after curative resection for colorectal cancer

**JD Howell[1], H Wotherspoon[1], E Leen[1], TC Cooke[1] and CS McArdle[2]**

[1]University Department of Surgery, Royal Infirmary, Glasgow G31 2ER, UK; [2]University Department of Surgery, Western General Hospital, Edinburgh EH4 2XU, UK

**Summary** Frequent liver imaging can detect liver metastases from colorectal cancer at an asymptomatic stage.

**Keywords:** colorectal cancer; follow-up; liver metastases

Recent studies have highlighted the lack of consensus among surgeons on the value of routine follow-up after apparently curative resection for colorectal cancer (Foster et al, 1987; Mella et al 1997). In theory, the aim of regular follow-up is to detect and treat recurrence at an early stage in an attempt to improve survival. Most studies have focused on the early detection of local recurrence amenable to surgery (Schiessel et al, 1986; Pollard et al, 1989; Camunas et al, 1991); however, there is increasing evidence that this approach is both ineffective and costly (Bruinvels et al, 1994; Biggs and Ballantyne, 1994; Virgo et al, 1995).

Because the commonest site of recurrent disease is liver, an alternative approach might be to focus on the early detection of liver metastases. The aim of the present study was to determine whether frequent liver imaging could detect liver metastases suitable for surgical or chemotherapeutic intervention at an asymptomatic stage.

### PATIENTS AND METHODS

Patients who had undergone potentially curative resections for colorectal cancer between 1990 and 1996 were followed at a dedicated clinic. Patients were reviewed at 3-month intervals for 2 years, and 6 monthly thereafter to 5 years. At each visit, the assessment included medical history, clinical examination, rectal examination when appropriate, full blood count, liver function tests and liver ultrasound. Computerized tomography (CT) scan of abdomen and pelvis was performed annually. Additional investigations were performed as appropriate.

Recurrences were classified as either locoregional or distant metastases. If patients had tumour-related symptoms at the time of detection, they were classified as having symptomatic recurrence. Patients who developed recurrence were investigated and treated as appropriate. Kaplan–Meier analysis was used to estimate survival curves.

*Received 19 December 1997*
*Revised 16 June 1998*
*Accepted 22 June 1998*

*Correspondence to:* CS McArdle

### RESULTS

One hundred and fifty-seven patients were included in the study. Sixty-eight (43%) were women and the mean age at diagnosis was 61 years. One hundred and two patients had colonic and 55 had rectal cancers; of these, six (4%) patients had Dukes' stage A tumours, 94 (60%) had Dukes' stage B and 57 (36%) had Dukes' stage C tumours.

The mean patient follow-up is 45 months. None of the patients with Dukes A tumours have recurred. To date, 18 (19%) patients with Dukes B tumours and 32 (56%) with Dukes C tumours have developed recurrent disease.

Fourteen (9%) patients developed local recurrence, a further eight (5%) developed local recurrence in conjunction with metastatic disease and 28 (56%) developed disseminated disease without evidence of local recurrence. Of the 36 patients with disseminated disease, the first site affected was the liver in 24 and the lungs in a further four. The remaining eight patients recurred at multiple sites. The majority (72%) of all recurrences were detected within 30 months of primary surgery.

Of the 22 patients (ten rectal, 12 colonic) who developed local recurrence, eight (36%) were asymptomatic at the time of detection. In contrast, of the 24 patients who developed histologically proven liver metastases as the initial site of recurrence, 21 (87%) were asymptomatic at the time of detection (Figure 1). Twelve

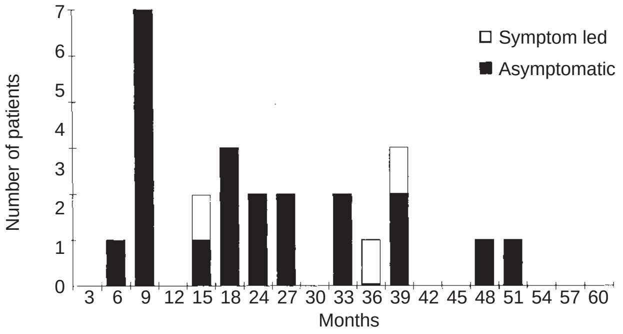

**Figure 1** Time to detection of liver metastases patients had metastases diagnosed initially by both ultrasound and CT scan and the remaining nine by CT alone. Two patients underwent hepatic resection for limited metastatic disease. Nineteen patients received chemotherapy; in nine of these, it was delivered by intra-arterial infusion. Patients with liver metastases detected at an asymptomatic stage had a median survival of 16 months (range 7–41 months) from the time of diagnosis, but survival in the group of patients with symptomatic metastases was less than 4 months.

### DISCUSSION

It has been traditional for surgeons to review their patients at regular intervals after potentially curative resection. It is, however, still not clear whether follow-up is of benefit. Enthusiasts believe that intensive follow-up and early intervention will lead to a reduction in the number of deaths from colorectal cancer (Sugarbaker et al, 1987; Safi and Beyer, 1993), whereas others point to the fact that the value of follow-up remains unproven (Cochrane et al, 1980; Ballantyne and Modlin, 1988).

Until recently, there were no randomized clinical trials, the best information being that from the meta-analysis performed by the Dutch group (Bruinvels et al, 1994). In that analysis, the results of seven non-randomized studies comparing routine and intensive follow-up, designed to detect local recurrence, in over 3000 patients were evaluated. More asymptomatic recurrences were detected and resected in the intensive follow-up group. However, there was no significant difference in survival. When the analysis was restricted to those studies that included carcinoembryonic antigen (CEA) measurements, there was an apparent 9% increase in 5-year survival in the intensively investigated group. The authors interpreted this data cautiously, recognizing that a number of biases were present.

Recently, three randomized trials comparing minimal with intensive follow-up have been reported (Makela et al, 1995; Ohlsson et al, 1995; Kjeldsen et al, 1997). In the largest of these studies, 600 patients were randomized to either 6 monthly follow-up or to follow-up visits at 5 and 10 years (Kjeldsen et al, 1997). Investigations consisted of history and clinical examination, full blood count and liver enzymes, faecal occult blood, chest radiograph and colonoscopy. No routine liver imaging was performed. Recurrence rates were similar in both groups but the tumour recurrences in the intensive group were detected on average 9 months earlier, often at an asymptomatic stage. Subsequently, more of these patients underwent reoperation with curative intent. There was, however, no difference in overall or cancer-related survival between the two groups. The authors concluded that an intensive follow-up plan is not justified considering the small proportion of patients reoperated on for cure and the fact that no survival advantage was detected.

An alternative approach has been evaluated by Northover et al (1994). Patients undergoing potentially curative surgery were randomized to an active intervention group or a control group. CEA was measured in all patients at frequent intervals. In the active intervention group, a rising CEA prompted further investigation, including second-look laparotomy if appropriate. Preliminary analysis has shown no difference in survival between the two groups.

It may be that the above studies were based on a false premise, namely that intensive follow-up would identify local recurrence at a stage when it would be amenable to further potentially curative surgery. However, the results of the above studies have shown that although intensive follow-up detects more local recurrences at an asymptomatic stage resulting in more reoperations, there is no difference in overall or disease-related survival.

Follow-up designed to detect asymptomatic liver metastases may be more effective. In our study, 21 of the 24 patients who developed liver metastases were detected at an asymptomatic stage; two underwent liver resection and 19 received chemotherapy. It has been estimated that 600 patients per year in the UK would be suitable for resection (Allen-Mersh, 1989); however, the number of resections performed is considerably less. It is worth noting that in the contemporary studies of liver resection, mortality is usually less than 5% and approximately 35% of patients survive 5 years (Ballantye, 1993). These survival figures are comparable or better than the results obtained after primary surgery for many types of gastrointestinal surgery. Furthermore, recent studies have shown that patients with disseminated disease receiving systemic chemotherapy at an asymptomatic stage had higher response rates, better quality of life and improved survival compared with those patients in whom the administration of chemotherapy was delayed until the patients became symptomatic (Nordic Gastrointestinal Tumor Adjuvant Therapy Group, 1992). Therefore, if liver metastases were diagnosed in more patients at a point when they would be amenable to treatment, either resection or chemotherapy, more long-term survivors might be anticipated.

Perhaps the time has come to readdress the question of follow-up. In addition to those investigations designed to detect loco-regional recurrence, future studies should include intensive liver imaging during the first 3 years, with a view to identifying patients suitable for either resection of liver secondaries or chemotherapy. Further randomized trials comparing intensive with minimal follow-up are currently being discussed at national and international level.

### REFERENCES

Allen-Mersh TG (1989) Colorectal liver metastases in ‘no treatment’ still the best? *J Roy Soc Med* **82**: 2–3

Ballantyne GH (1993) Surgical treatment of liver metastases in patients with colorectal cancer. *Cancer* **71**: 4252–4266

Ballantyne GH and Modlin IM (1988) Postoperative follow-up for colorectal cancer: Who are we kidding? *J Clin Gastroenterol* **10**: 359–364

Biggs CG and Ballantyne GH (1994) Sensitivity versus cost effectiveness in postoperative follow-up for colorectal cancer. *Curr Opinion Gen Surg* 94–102

Bruinvels DJ, Stiggelbout AM, Kievut J, Van Houwelingen H, Habbema DF and Van de Velde C (1994) Follow-up of patients with colorectal cancer: a meta-analysis. *Ann Surg* **219**: 174–182

Camunas J, Enriquez JM, Devesa JM, Morales V and Millan I (1991) Value of follow-up in the management of recurrent colorectal cancer. *Eur J Surg Oncol* **17**: 530–535

Cochrane JPS, Williams JT, Faber RG and Slack WW (1980) Value of outpatient follow-up after curative surgery for carcinoma of the large bowel. *Br Med J* **280**: 593–595

Foster ME, Hill J and Leaper DJ (1987) Follow-up after colorectal cancer – current practice in Wales and South West England. *Int J Colorectal Dis* **2**: 118–119

Kjeldsen B, Kronberg O, Fenger C and Jorgensen O (1997) A prospective randomised study of follow-up after radical surgery for colorectal cancer. *Br J Surg* **84**: 666–669

Makela JT, Laitinen SO and Kairaluoma MI (1995) Five year follow-up after radical surgery for colorectal cancer: results of a prospective randomised trial. *Arch Surg* **130**: 1062–1067

Mella J, Datta SN, Biffin A, Radcliffe AG, Steele RJC and Stamatakis JD (1997) Surgeons follow-up practice after resection of colorectal cancer. *Ann R Coll Surg England* **79**: 206–209

Nordic Gastrointestinal Tumour Adjuvant Therapy Group (1992) Expectancy or primary chemotherapy in patients with advanced asymptomatic colorectal cancer: a randomised trial. *J Clin Oncol* **10**: 904–911

*© Cancer Research Campaign 1999*

Northover J, Houghton J and Lennon T (1994) CEA to detect recurrence of colon cancer. *JAMA* **272**: 31

Ohlsson B, Breland U, Ekberg H, Graffner H and Tranberg K (1995) Follow-up after curative surgery for colorectal carcinoma: randomised comparison with no follow-up. *Dis Colon Rectum* **38**: 619–626

Pollard SG, Macfarlane R and Everett WG (1989) Surgery for recurrent colorectal carcinoma – is it worthwhile? *Ann R Coll Surg England* **71**: 293–298

Safi F and Beyer HG (1993) The value of follow-up after curative surgery of colorectal carcinoma. *Cancer Detect Prev* **17**: 417–424

Schiessel R, Wunderlich M and Herbst F (1986) Local recurrence of colorectal cancer: effect of early detection and aggressive surgery. *Br J Surg* **73**: 342–344

Sugarbaker PH, Gianola FJ, Dwyer A and Neuman NR (1987) A simplified plan for follow-up of patients with colon and rectal cancer supported by prospective studies of laboratory and radiologic test results. *Surgery* **102**: 79–87

Virgo KS, Verava AM, Longo W, McKirgen LW and Johnson F (1995) Cost of patient follow-up after potentially curative colorectal cancer treatment. *JAMA* **273**: 1837–1841

*© Cancer Research Campaign 1999*
